# Increased detectability of Apolipoprotein E (ApoE) in cerebrospinal fluid samples of dogs with peracute and acute central nervous system lesions

**DOI:** 10.3389/fvets.2026.1901246

**Published:** 2026-09-03

**Authors:** Michelle Prielipp, Eva-Maria Packeiser, Andrea Bathen-Nöthen, Reinhard Mischke, Andrea Tipold

**Affiliations:** 1 Department of Small Animal Medicine and Surgery, University of Veterinary Medicine Hannover, Foundation, Hannover, Germany; 2 Veterinary Practice Dr. Med. Vet. Andrea Bathen-Nöthen, Small Animal Neurology, Cologne, Germany

**Keywords:** ApoE, Apolipoprotein E, C-reactive protein, CRP, CSF, dog, neurology

## Abstract

**Introduction:**

Apolipoprotein E (ApoE), secreted by astrocytes and microglia after neuronal injury, is involved in neuronal regeneration and modulation of inflammatory processes. Inverse associations between ApoE and the proinflammatory mediator C-reactive protein (CRP) are described in mice, but knowledge in dogs is limited.

**Methods:**

This study investigated ApoE levels in cerebrospinal fluid (CSF) from 141 dogs with central nervous system (CNS) diseases, including idiopathic epilepsy (*n* = 40), intervertebral disc herniation (IVDH, *n* = 40), meningoencephalitis of unknown origin (MUO, *n* = 30), degenerative myelopathy (*n* = 31), and 10 healthy controls. ApoE concentrations were measured by ELISA. CRP levels in CSF and paired serum samples were determined by immunoassay. Correlations between ApoE, clinical parameters and CRP were evaluated. We hypothesized elevated CSF ApoE in acute CNS diseases and low or undetectable levels in chronic conditions and a negative correlation between ApoE and CRP.

**Results:**

ApoE was detected in 66/151 cases, of which 20 showed quantifiable levels, exclusively in diseased dogs. While dogs with peracute IVDH or high seizure frequency were significantly associated with detected ApoE, MUO cases with multiple lesions only had a trend for measurable ApoE levels. Contrary to our hypothesis, a significant positive correlation between ApoE and CRP in CSF was found, suggesting a potential modulatory role of ApoE in neuroinflammation.

**Discussion:**

In conclusion, ApoE may play a role particularly during peracute and acute disease stages, while low CSF ApoE may reflect regenerative processes in affected neuronal tissue.

## 1 Introduction

Neurological diseases in dogs are becoming increasingly common in veterinary practice and are frequently associated with neuronal damage and inflammatory processes. The most common neurological diseases in dogs include idiopathic epilepsy (IE), intervertebral disc herniation (IVDH) followed by inflammatory diseases of the central nervous system (CNS) with meningoencephalomyelitis of unknown origin (MUO) accounting for the majority of this disease group (1–4). Validated biomarkers are crucial for both diagnostic and prognostic assessment of diseases and for the early initiation of appropriate therapies, as they enable the identification, characterization, and monitoring of their occurrence, severity, and progression (5). They are particularly important in case of severe neurological deficits and diseases with a strong impact on the quality of life of affected dogs and their owners in order to enable an assessment of the prognosis.

Apolipoprotein E (ApoE) has been investigated in both cerebrospinal fluid (CSF) and neuronal tissue in various human and murine studies as a biomarker for the prognosis of neurological diseases such as Alzheimer's disease (6–10). As a major component of lipoprotein particles, it is essential for lipoprotein and lipid metabolism (11, 12). After neuronal injury, ApoE is primarily secreted and lipidated by astrocytes, but also by microglia and neurons and subsequently migrates into neurons (13, 14). The supply of astrocytic cholesterol to neurons by ApoE-containing lipoproteins plays a crucial role in synaptogenesis and regeneration following neuronal damage (11, 15, 16). ApoE-deficient mice show synaptic and dendritic changes, increased loss of synapses and neuronal apoptosis (17–19). Up to now, the only study conducted in dogs found an altered lipid metabolism in ApoE-deficient individuals (20, 21).

ApoE is upregulated in reactive astrocytes and neurons in both laboratory rodents and humans shortly after CNS injury, where it exerts immunomodulatory effects by modulating microglial and astrocytic responses to inflammation (13, 22–24). ApoE-deficient mice express more proinflammatory cytokines compared to wild-type mice (19, 23).

An important inflammatory mediator is C-reactive protein (CRP) which is synthesized by hepatocytes but also in neurones, microglia and astrocytes in the CNS, in response to proinflammatory cytokines following traumatic or inflammatory insults (25–27). Empirical studies indicate an inverse relationship between ApoE and CRP, supporting the role of ApoE as a potential immune regulator in inflammatory processes (21, 28). Although IE and IVDH are not primarily inflammatory diseases, there is evidence for the involvement of inflammatory processes (29, 30).

Current data on ApoE concentrations in the CSF of neurologically diseased dogs are lacking. The extracellular space of the brain and spinal cord is in direct contact with the CSF, meaning biochemical processes of the central nervous system can be reflected in this fluid (31). Therefore, ApoE in CSF may be represent a potential biomarker of active regeneration processes in various forms of brain and spinal cord damage.

This study aimed to investigate the detection of ApoE in the CSF of dogs with CNS diseases and to examine the relationship between ApoE and the development of primary non-inflammatory, inflammatory and degenerative CNS diseases in dogs. Additionally, the relationship between ApoE concentrations in the CSF and CRP values in both CSF and serum samples of dogs with neurological diseases mentioned was investigated. We hypothesized that ApoE is detectable in increased concentrations in the CSF in acute neurological diseases in dogs, whereas in chronic processes it is only detectable in low concentrations or remains below the detection limit. Furthermore, we hypothesized that there is a negative correlation between ApoE in CSF and CRP in CSF and serum in neurological diseases in dogs.

## 2 Material and methods

### 2.1 Subjects, selection criteria, diagnostic procedures

In this retrospective bicentric study, 141 canine patients with a neurological disease presented between January 2012 and December 2024—either to the Department of Small Animal Medicine and Surgery, University of Veterinary Medicine Hannover, Foundation (Hannover, Germany) (*n* = 132), or to the veterinary referral practice Vetneuro, Dr. med. vet. Andrea Bathen-Nöthen (Cologne, Germany) (*n* = 9)—were included. A statistical power of 81.5% was calculated using the Kruskal-Wallis test, based on a control group size of 10 animals. Ethical approval was granted by the Lower Saxony State Office for Consumer Protection and Food Safety (LAVES), Germany (approval number: 33.8-42502-04-24-00553). CSF and serum were collected from 10 clinically and neurologically healthy Beagle dogs. Sampling was performed under general anesthesia and in accordance with national and European animal welfare regulations (Directive 2010/63/EU). All samples were stored after sampling at −80 °C.

**Figure 1:**
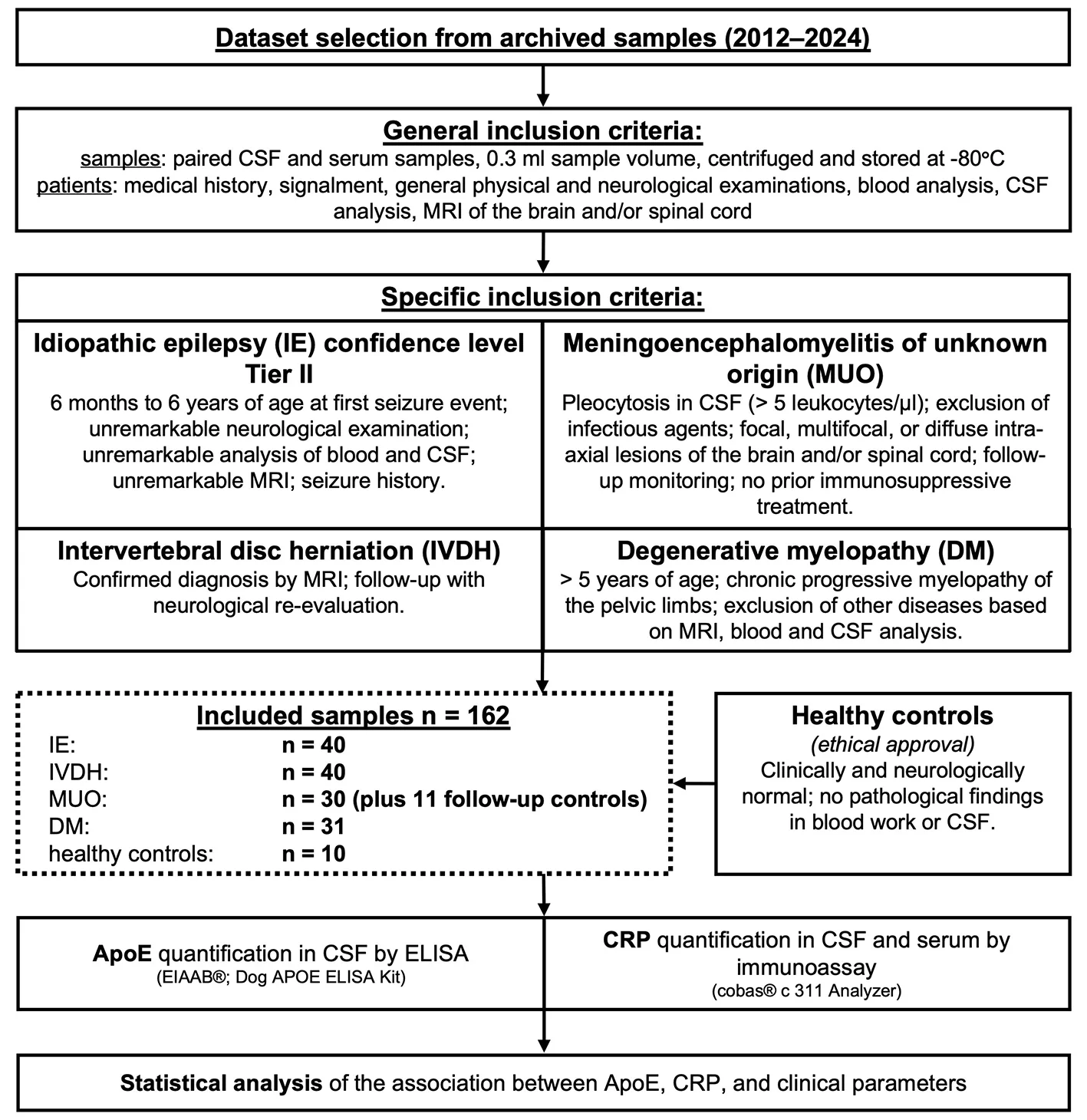
Study protocol including general and specific inclusion criteria. A total of 162 paired CSF and serum samples, including 11 existing follow-up controls from dogs with MUO, met the inclusion criteria. Signalment comprised breed, sex, neuter status, date of birth, and body weight. Hematological and biochemical analyses included complete blood count with differential, clinical chemistry, and immunoglobulin A (IgA). CSF analysis encompassed total protein, albumin, cell count with differential, and IgA. ELISA, enzyme-linked immunoassay; MRI, magnetic resonance imaging; ApoE, Apolipoprotein E; CRP, C-reactive protein; CSF, cerebrospinal fluid.

The study population was selected from a curated digital in-house archive of preserved samples. Figure 1 illustrates the study protocol, including general and specific criteria. Dogs diagnosed with one of the following four diseases, based on established definitions and classifications from the literature, were included: IE confidence level tier II (32), IVDH (33), MUO (34) and degenerative myelopathy (DM) (35). A total of 162 paired CSF and serum samples, including 11 existing follow-up controls from dogs with MUO and 10 healthy controls, met the inclusion criteria. All patient samples were used as residuals of previous routine diagnostic procedures with the owners' written consent. Clinical diagnoses were established based on a combination of clinical and neurological examination, blood analysis, magnetic resonance imaging (MRI) of the brain and/or spinal cord, CSF analysis and, if available, (histo-) pathological examination of a biopsy sample or the entire carcass. Relevant clinical data were extracted from the clinical management software easyVET (Veterinärmedizinisches Dienstleistungszentrum [VetZ] GmbH, Isernhagen, Germany). Data and measurement results for each patient were systematically recorded and managed using spreadsheet software (MS Excel 2024, Microsoft Corporation, Redmond, USA).

### 2.2 Measurement of ApoE in CSF with an enzyme-linked immunosorbent assay (ELISA)

A total of 162 CSF samples were analyzed in duplicates for ApoE concentrations using a commercially available ELISA kit (Dog APOE/Apolipoprotein E ELISA Kit, No. E0704d, EIAAB Science INC, Wuhan, China) from the same batch, according to manufacturer's protocols. Preliminary own experiments revealed no measurable ApoE concentrations in serum (unpublished data); therefore, ApoE quantification was limited to CSF in the main study. According to our pilot-testing results all samples were diluted 1:2 using the sample diluent provided with the kit.

According to the manufacturer's specifications, the lower limit of quantification (LLOQ) for undiluted samples was 1.56 ng/ml, corresponding to 3.12 ng/ml after dilution. The lower limit of detection was 0.81 ng/ml (undiluted), corresponding to 1.62 ng/ml after dilution. Plates were analyzed using spectrophotometric measurements at 450 nm using a Synergy™ 2 Multi-Mode Microplate Reader (BioTek Instruments, Inc., Bad Friedrichshall, Germany). The standard curve was generated by inputting the target ApoE concentrations in BioTek Gen5 Version 2.0 software (BioTek Instruments, Inc., Winooski, VT, USA) and applying point-to-point interpolation. Sample absorbance values were converted to ng/ml based on the standard curve and adjusted for the dilution factor. The arithmetic mean of duplicates was calculated to determine the mean ApoE concentration (MApoE) for each sample.

Assay precision was evaluated using two control samples: a 12.5 ng/ml ApoE solution prepared from assay reagents and a pooled CSF sample (consisting of 40 leftover diagnostic samples from undefined neurological diseases, without known concentration) diluted 1:2. All control samples were measured in duplicate. In total, 26 respectively 24 measurements for the 12.5 ng/ml ApoE solution and diluted CSF pool were obtained across nine ELISA plates.

Inter-assay precision was determined by calculating the mean of duplicate measurements for each control sample on each plate. The coefficient of variation (CV) was then calculated across plates as the standard deviation (SD) of these plate-specific means divided by their overall arithmetic mean.

Intra-assay precision was evaluated on a single ELISA plate using five independent replicate measurements of each control sample, each analyzed in duplicate. The mean of each duplicate pair was used to calculate the SD and CV.

### 2.3 Measurement of CRP and biochemistry in serum and CSF

All measurements were performed in the institution's in-house laboratory. White blood cell count was analyzed using the hematology system ADVIA^®^ 2120i (Siemens Healthineers, Erlangen, Germany). Blood clinical chemical analyses (total protein and albumin concentrations) were performed with the Cobas^®^ c 311 analyzer (Roche Diagnostics, F. Hoffmann-La Roche AG, Basel, Switzerland) using reagents, standards and control material provided by the manufacturer. This analyzer was also utilized to measure CRP in blood serum and CSF (paired samples) using an immunoassay.

CSF analysis (cell count, and protein concentrations) was performed according to standard laboratory protocols.

IgA in paired serum and CSF samples was quantified by ELISA, as previously described (36).

### 2.4 Statistical analysis

Statistical analyses and graphical visualizations were conducted using SAS^®^ Studio 3.81 (Enterprise Edition; SAS Institute GmbH, Heidelberg, Germany) and GraphPad Prism^®^ (version 9.5.1 GraphPad Software, LLC, Boston, MA, USA, RRID:SCR_002798) after consulting with the Institute of Biometry, Epidemiology and Information Processing at the University of Veterinary Medicine Hannover. Statistically significant was defined as p < 0.05.

The presence of ApoE in CSF was compared between study groups and analyzed in relation to clinical parameters, laboratory findings and CRP in serum and CSF.

Primary analyses were based on a qualitative classification of ApoE concentrations (CApoE) according to the assay's limited analytical sensitivity. Concentrations below the LLOQ could not be reliably quantified and were therefore treated as left-censored. Based on the LLOQ, CApoE was categorized into three groups: C0 (undetectable ApoE levels; 0 ng/ml), C1 (detectable but non-quantifiable concentrations; > 0 to < 3.12 ng/ml), and C2 (quantifiable concentrations; ≥ 3.12 ng/ml). In addition to the primary categorical analyses, quantitative analysis of MApoE was performed as complementary exploratory analyses. For this purpose, samples classified as C0 and C1 were assigned fixed values of 0.01 ng/ml and 3.11 ng/ml, respectively, to enable approximate numerical comparison. These assigned values were used exclusively for the exploratory quantitative MApoE analyses. A comparison between diseased and control animals was performed using the Mann-Whitney U test. Group differences across all study groups were initially assessed using the Kruskal-Wallis test followed by Dunn's *post hoc* test for pairwise comparisons.

Normal distribution of CRP values was assessed using the Shapiro-Wilk test. The CRP values of the diseased dogs deviated significantly from a normal distribution in both serum and CSF. In healthy controls, serum CRP were normally distributed, whereas CSF CRP deviated from normality. Accordingly, non-parametric methods were applied to compare CRP levels in serum and CSF between diseased and healthy dogs (Mann-Whitney U test) and among study groups (Kruskal-Wallis test; Dunn's test as *post hoc* test). Spearman correlation was used to compare CRP in Serum und CRP in CSF.

Descriptive statistics (frequencies for categorical, dispersion measures for continuous numerical parameters) were calculated for the full cohort and subgroups (control group, IE, IVDH, MUO, DM).

Ordinal logistic regression (Wald test) was used to analyze the relationship between CApoE and study population groups, clinical parameters, laboratory findings and CRP levels in CSF and serum.

## 3 Results

### 3.1 Descriptive population data

Population characteristics of healthy controls (*n* = 10) and patients (*n* = 141) are summarized in Tables 1, 2. Among the diseased dogs, males were significantly overrepresented compared to females (93 vs. 48; *p* < 0.01, Fisher's exact test); sex and neuter status were unavailable for one dog. The most common breeds are described in Table 1. The predominant seizure type of dogs with IE were generalized seizures (77.5%), with cluster seizures occurring most frequently (52.5%). Mean seizure frequency over the preceding month was 7 episodes (range: 0–52 episodes). In dogs diagnosed with IVDH, most lesions were located in the cervical region (47.5%), with Hansen type I extrusion being the most frequent diagnosis (52.5%), followed by acute non-compressive nucleus pulposus extrusion (ANNPE) and hydrated nucleus pulposus extrusion (HNPE), each accounting for 20.0%. Dogs diagnosed with MUO exhibited multifocal MRI lesions in 70.0% of cases, and epileptic seizures were reported in 26.7% of dogs, with generalized and/or cluster seizures most frequently observed. Follow-up CSF analysis was available in 11 of 30 dogs (37.0 %) after initiation of immunosuppressive therapy; 90.9 % showed clinical improvement in addition to milder laboratory CSF changes in comparison to the first examination. Smaller MRI lesions were detected in 9 cases with MUO. Dogs diagnosed with DM were predominantly classified as Sharp and Wheeler grade 2 (80.6%), presented with paraparesis (83.9%), and showed no relevant findings on MRI (64.5%); superoxide dismutase 1 (SOD1) genotyping was performed in 54.8% of cases, of which 41.9% were homozygous for the risk allele.

**Table 1:** Demographic characteristics of healthy control dogs and dogs diagnosed with CNS diseases.

Study group	*n*	Age, months	Sex (female; male)	Neutered (female; male)	Body weight, kg	Breed (*n*)
Healthy controls	10	27.0 (27.0–28.0)	5; 5	0; 0	12.0 (12.8–13.7)	Beagle (10).
IE	40	37.5 (23.5–55.3)	11; 29	5; 9	30,3 (22.8–39.4)	Mixed breed (10); Labrador Retriever (6); Australian Shepherd, Border Collie (3 each); Great Dane, Golden Retriever, Presa Canario, Swiss mountain dog, Siberian Husky (2 each); Akita, Bolonka Zwetna, Dogue de Bordeaux, German Hound, Cane Corso, German Pinscher, German Shepherd, Standard Poodle (1 each).
IVDH	40	80.0 (62.0–108.3)	15; 25	8; 11	14.4 (9.8–23.1)	Mixed breed (12); French Bulldog (5); Border Collie, Dachshund, Jack Russell Terrier (2 each); American Bulldog, Bernese Mountain Dog, Cocker Spaniel, Long-haired Dachshund, Dalmatian, Great Dane, English Bulldog, Irish Terrier, Small Munsterlander, Labrador Retriever, Maltese, Pointer, German Shepherd, Staffordshire Bull Terrier, Yorkshire Terrier, Pomeranian (1 each).
MUO	30	54.5 (35.8–95.0)	12; 18	3; 6	21.5 (9.4–30.0)	Mixed breed (6); Labrador Retriever (3); Chihuahua, Golden Retriever, Pug (2 each); Australian Kelpie, Australian Shepherd, Belgian Shepherd, German Shepherd, Elo, Eurasier, French Bulldog, Gordon Setter, Hanoverian Scent hound, Havanese, Hovawart, Maltese, Rhodesian Ridgeback, Shih Tzu, West Highland White Terrier (1 each).
DM	31	108.3 (117.5–129.0)	9; 21 1 unknown	7; 9 1 unknown	31.0 (22.9–35.7)	Mixed breed (10); Hovawart (3); Bernese Mountain Dog, Chodský Pes, German Shepherd, Podenco (2 each); Boxer, Gordon Setter, Standard Poodle, Kuvasz, Rough Collie, Parson Russell Terrier, Puggle, Staffordshire Bull Terrier (1 each).

Numerical data are shown as median [interquartile range (Q1–Q3)], categorical variables as counts.

**Table 2:** Clinical and laboratory findings of dogs diagnosed with CNS diseases and their association with CApoE.

Study group	Clinical parameter	Value	Significant relationship with CApoE
Whole study cohort	Serum CRP (mg/dl)	See Figure 3A	Not significant
	CSF CRP (mg/dl)	See Figure 3B	Significant (*p* = 0.02; OR = 0.81)^*^
IE	Disease duration in days	213 (0–1460)	Not significant
	Interval from last seizure to sampling in days	11 (0–66)	Not significant (< 24 h vs. >24 h); trend toward higher likelihood of detectable CSF ApoE with shorter seizure-to-sampling interval (0–12 days).
	Phenotype of epileptic seizures	Focal (5.0%); focal and generalized (17.5%); generalized (77.5%)	Not significant
	Severity of epileptic seizures	Single seizure events (32.5%); cluster seizures (52.5%); status epilepticus and cluster seizures (15.0%)	Trend for C2 for dogs with cluster seizures and/or status epilepticus
	Frequency of epileptic seizures last months	7 (0–52)	Significant (*p* = 0.04; OR = 0.935)^*^
	Frequency of epileptic seizures last 6 months	14 (1–121)	Not significant
	Number of ASD	0 (47.5%); 1 (30.0%); 2 (17.5%); > 2 (5.0%)	Not significant
	Application of MCT oil	Yes (20.0%); no (80.0%)	Not significant
	Serum CRP (mg/dl)	See Figure 3A	Not significant
	CSF CRP (mg/dl)	See Figure 3B	Not significant
IVDH	Disease duration in days	16 (0–183)	Not significant
	Degree according to Sharp and Wheeler (2005)	Grade 1 (10.0%); grade 2 (45.0%); grade 3 (27.5%); grade 4 (15.0%); grade 5 (2.5%)	Not significant
	Neurological impairment pattern	Paraparesis/-plegia (45.0%); tetraparesis/-plegia (37.5%); spinal ataxia (5.0%); hemiparesis (2.5%)	Not significant
	MRI-based localization of IVDH	Cervical (47.5%); cervicothoracic (7.5%); thoracal (20.0%); thoracolumbar (15.0%); lumbar (10.0%)	Not significant
	MRI-based type of IVDH	ANNPE (20.0%); HNPE (20.0%); Hansen type I (52.5%); Hansen type II (7.5%)	Significant (*p* = 0.04; OR = 2.059)^*^
	Therapy received	Conservative (55.0%); surgery (45.0%)	Not significant
	Neurological development	Improved (77.5%); no change (12.5%); deteriorated (10.0%)	Not significant
	Days until neurologically improvement	28 (1–187)	Not significant
	Serum CRP (mg/dl)	see Figure 3A	not significant
	CSF CRP (mg/dl)	see Figure 3B	Significant (*p* = 0.01; OR = 0.579)^*^
MUO	Disease duration in days	95 (0–2159)	Not significant
	Behavior	Unremarkable (63.3%); altered (36.7%)	Not significant
	Consciousness	Unremarkable (70.0%); altered (30.0%)	Not significant
	Gait	Unremarkable (13.3%); altered, ambulatory (63.3%); altered, non-ambulatory (23.4%)	Not significant
	Head posture	Unremarkable (43.7%); altered (56.3%)	Not significant
	Visus	Unremarkable (67.7%); restricted or blind (32.3%)	Not significant
	Epileptic seizures	No (73.3%); yes (26.7%)	Not significant
	Type of epileptic seizure	Focal (37.5%); focal and generalized (12.5%); generalized (50.0%)	Not significant
	Severity of epileptic seizures	Single seizure events (25.0%); cluster seizures (37.5%); status epilepticus and cluster seizures (37.5%)	Not significant
	Frequency of epileptic seizures last months	8 (0–21)	Not significant
	Frequency of epileptic seizures last 6 months	8 (0–21)	Not significant
	Number of ASD	0 (62.5%); 1 (3.0%); 2 (17.5%);	Not significant
	MRI-based lesion distribution on MRI	Focal (13.3%); multifocal (70.0%); diffuse (16.7%)	Trend for higher CApoE in multiple lesions compared to lesions limited to one location
	MRI-based localization of lesion	Cerebrum (16.7%); cerebellum (3.3%); brain stem (6.7%); ≥ 2 localization (73.3%)	Not significant
	Clinical improvement within the first 7 days after treatment initiation	Yes (86.7%);	Not significant
	MRI findings improvement	Yes (40.0%); no (3.3%); no re-examination (56.7%)	not significant
	Laboratory CSF findings improvement	Yes (50.0%); no (3.3%); no re-examination (46.7%)	Not significant
	Days until euthanasia	236 (8–913)	Not significant
	Serum CRP (mg/dl)	See Figure 3A	Not significant
	CSF CRP (mg/dl)	See Figure 3B	Not significant
DM	Disease duration in days	226 (15–730)	Not significant for < 6 months vs. > 6 months
	Degree according to Sharp and Wheeler	Grade 2 (80.6%); grade 3 (19.4%);	Not significant
	Neurological impairment pattern	Paraparesis (83.9%); tetraparesis (6.5%); spinal ataxia (9.7%);	Not significant
	MRI findings	Unremarkable (64.5%); alterations (35.5%)	Not significant
	SOD1 gene mutation status	Homozygous for risk allele (47.9%); heterozygous (carrier) (3.2%); homozygous for the wild-type allele (9.7%); no testing performed (45.2%)	Not significant
	Serum CRP (mg/dl)	See Figure 3A	Not significant
	CSF CRP (mg/dl)	See Figure 3B	Not significant

Numerical data are shown as mean (range), categorical data as percentages. Disease duration was defined as the time from onset of clinical signs to CSF sampling.

### 3.2 Control samples and intra- and interassay precision for ApoE determination

Assay precision was evaluated using a 12.5 ng/ml ApoE solution and a diluted CSF pool.

Intra-assay precision, defined as the variability of replicate measurements within a single ELISA plate, showed CVs of 9.0% for the 12.5 ng/ml ApoE solution and 8.9% for the CSF pool.

Inter-assay precision, reflecting variability between different ELISA plates and experimental runs, resulted in CVs of 35.21% for the 12.5 ng/ml ApoE solution and 31.67% for the CSF pool.

The ApoE control solution yielded a mean measured concentration of 10.06 ng/ml (range: 4.47–14.47 ng/ml), while the CSF pool showed a mean concentration of 9.21 ng/ml (range: 3.85–13.30 ng/ml).

### 3.3 ApoE detectability in CSF and its association with clinical parameters

Detailed results for every parameter and its association with ApoE are shown in Table 2.

**Figure 2:**
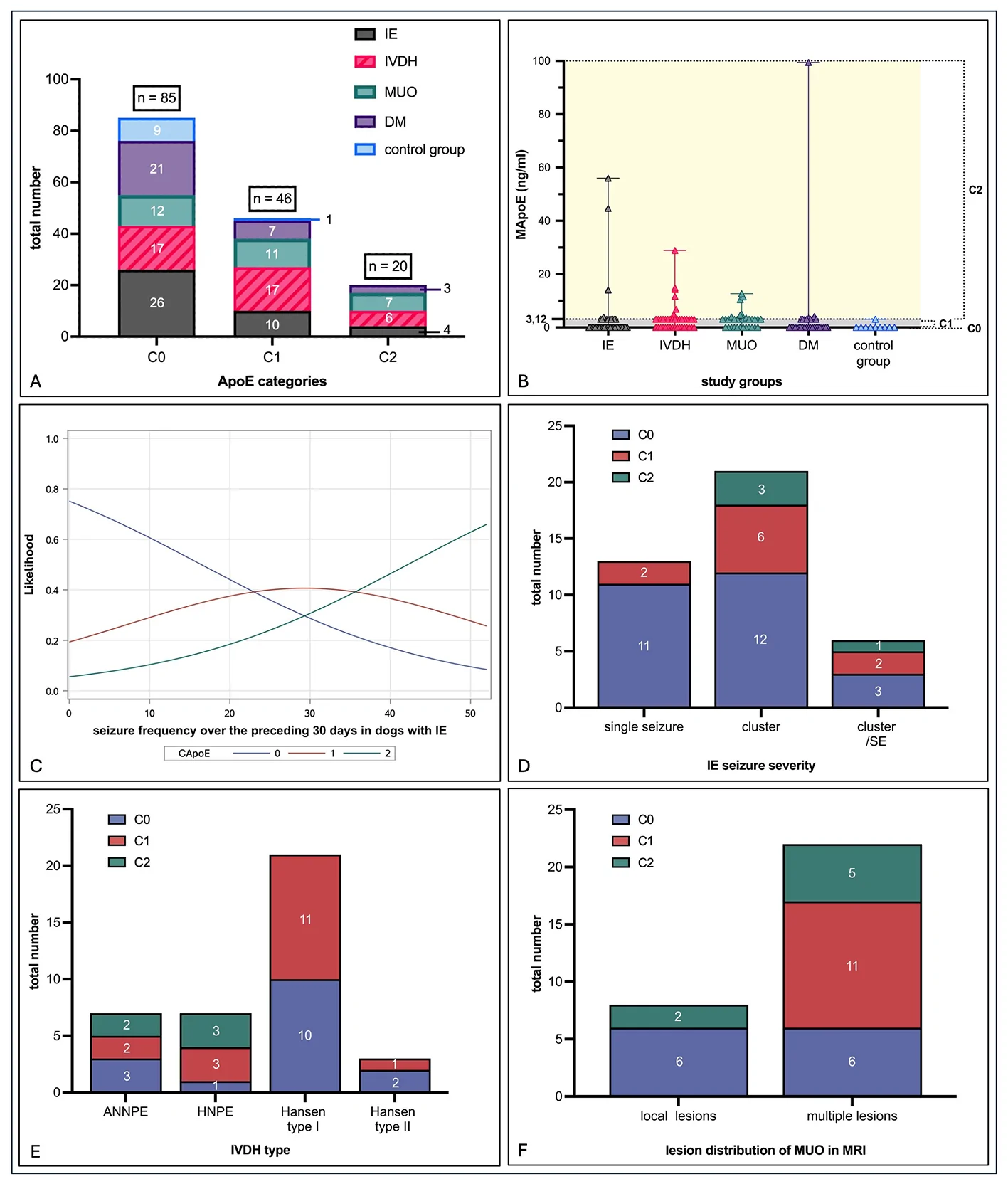
Detectability and distribution of ApoE in CSF of healthy control dogs and dogs diagnosed with idiopathic epilepsy (IE), intervertebral disc herniation (IVDH), meningoencephalitis of unknown origin (MUO), or degenerative myelopathy (DM). **(A)** Stacked bar plot showing the distribution of ApoE categories (CApoE) across study groups, with one healthy control classified as C1; C0 = 0 ng/ml—blue, C > 0 to > 3.12 ng/ml—red, C2 ≥3.12 ng/ml—green. **(B)** Scatter plot of ApoE concentrations in the study population and healthy controls. Triangles represent the mean of duplicate measurements (MApoE, ng/ml). Horizontal dotted lines indicate the CApoE cut-off values: 0 ng/ml (C0) and 3.12 ng/ml (threshold between C1 and C2). The shaded gray and yellow areas represent C1 and C2, respectively. There were no statistically significant differences in the pairwise comparisons between individual groups. **(C)** Estimated probabilities of CApoE (y-axis) in relation to seizure frequency over the preceding 30 days in dogs with IE (x-axis). Lines represent predicted probabilities across the range of number of seizures, with color coding corresponding to ApoE concentrations **(C)**: not detectable (0, blue), C = 0 ng/ml; detectable but not quantifiable (1, red), C > 0 to < 3.12 ng/ml; and quantifiable (2, green), C ≥ 3.12 ng/ml. A significant association between seizure frequency over the preceding 30 days and CApoE was observed (*p* = 0.04; OR = 0.935; ordinal logistic regression, Wald test). **(D–F)** Stacked bar plots showing of the distribution of CApoE across subgroups. **(D)** IE cases stratified by seizure severity [single seizure, cluster seizures, cluster seizures with status epilepticus (SE)]. Dogs with cluster seizures and cluster/SE more frequently exhibited quantifiable ApoE concentrations without statistically significance. **(E)** Dogs with IVDH stratified across different IVDH types (acute non-compressive nucleus pulposus extrusion [ANNPE] and hydrated nucleus pulposus extrusion [HNPE]), Hansen type I extrusion, and Hansen type II protrusion. Only dogs with ANNPE or HNPE were classified as C2 (*p* = 0.04; ordinal logistic regression, Wald test). **(F)** Dogs with MUO stratified by lesion distribution on MRI (local vs. multiple lesions), while dogs with multiple lesions tended to have a higher CApoE compared with those with a single lesion; however, this difference was not statistically significant. Numbers within the bars indicate the number of dogs in each CApoE. Color coding corresponds to ApoE concentrations as defined above.

ApoE was detectable in the CSF of 66 out of 151 dogs (43.7%), including 20 dogs (13.3%) classified as C2 (exclusively from diseased dogs) and 46 dogs (30.5%) classified as C1 (including 5 diseased dogs and one healthy control) (Figure 2A). 85 dogs (56.3%) were classified as C0 due to undetectable ApoE (including 76 diseased dogs and 9 healthy controls). The overall mean MApoE within C2 was 17.95 ng/ml (range: 3.53–99.42 ng/ml) with the highest value observed in the DM group (Figure 2B). MApoE was significantly increased in diseased dogs compared to healthy controls (*p* = 0.03; Mann-Whitney U test) and showed a significant overall difference among the subgroups, including healthy controls (*p* = 0.02; Kruskal-Wallis test). However, none of the pairwise comparisons between individual groups reached statistical significance (Figure 2B). A sensitivity analysis excluding the highest MApoE value in the DM group yielded comparable results. Both the comparison between diseased and healthy dogs and the overall group comparison remained statistically significant (*p* = 0.03 and *p* = 0.01, respectively), while no pairwise comparisons between individual groups reached statistical significance.

The percentage of results in C1 und C2 were significantly higher in diseased dogs (66/141; 43.7%) compared to the healthy control dogs (1/10; 10%) (*p* = 0.04; Fisher's exact test). The odds of being healthy were 7.7 times higher in the absence of a detectable ApoE in the CSF. CApoE did not differ significantly between disease groups (IE, IVDH, MUO, DM) (*p* = 0.91; OR = 0.983; ordinal logistic regression [Wald test]).

The association between study population characteristics and CApoE showed no significant correlation for age, sex, or body weight either within the overall study population or across the study groups.

In dogs with IE, a significant association was identified between CApoE and seizure frequency over the preceding 30 days (p = 0.04; OR = 0.935; ordinal logistic regression [Wald test]), indicating that higher seizure frequencies were associated with an increased likelihood of classification into a higher CApoE (Figure 2C). No significant association was found between the time since the last seizure (< 24 h vs. > 24 h) and CApoE. However, ApoE was more likely detected in the CSF, when the time interval between CSF sampling and the most recent seizure was short (0 to 12 days). Notably, 11 dogs out of 40 experienced their last seizure more than 12 days prior to sampling and exhibited no detectable ApoE levels in the CSF. Although no significant association was found between seizure severity and CApoE classification, there was a trend suggesting a higher likelihood of C2 in dogs with cluster seizures and/or status epilepticus compared with dogs presenting with single seizures. Consistently, C2 classification was exclusively observed in dogs with cluster seizures and/or status epilepticus, whereas dogs with single seizures were confined to categories C1 and C0 (Figure 2D).

In dogs with IVDH, all dogs classified as C2 were diagnosed with ANNPE or HNPE, whereas dogs with Hansen type I extrusion or Hansen type II protrusion were exclusively assigned to categories C1 or C0 (Figure 2E). Consistent with this distribution, dogs with ANNPE or HNPE had a significantly higher estimated probability of being classified as C2 compared to dogs with Hansen type I extrusion or Hansen type II protrusion (*p* = 0.04; OR = 2.059; ordinal logistic regression [Wald test]). In addition, dogs with ANNPE or HNPE showed a shorter duration of clinical signs prior to presentation (median 1 day, range 0–14 days) compared to dogs with Hansen type I extrusion or Hansen type II protrusion (median 5 days, range 0–183 days), a difference that was also statistically significant (*p* = 0.0009; Mann-Whitney U test). Severe deficits (Sharp and Wheeler grade 4 and 5) were more frequently observed in C2 compared to C0 and C1, but without statistical significance.

In dogs with MUO, dogs with multiple lesions (22/30; 73.3%) had a higher CApoE compared to dogs with lesions limited to one location (8/30; 26.7%) but without statistical significance (Figure 2F). In MUO controls, seven dogs (72%) remained in the same CApoE following initiation of immunosuppressive therapy, while three dogs (27%) shifted to a lower category and one dog (9.0%) shifted to a higher category. An increase in ApoE concentration post-therapy was observed in two dogs (18%), whereas a measurable decrease was detected in two dogs (18%).

In dogs with DM, none of the clinical parameter showed a significant association with CApoE.

### 3.4 CRP detectability and association with ApoE

**Figure 3:**
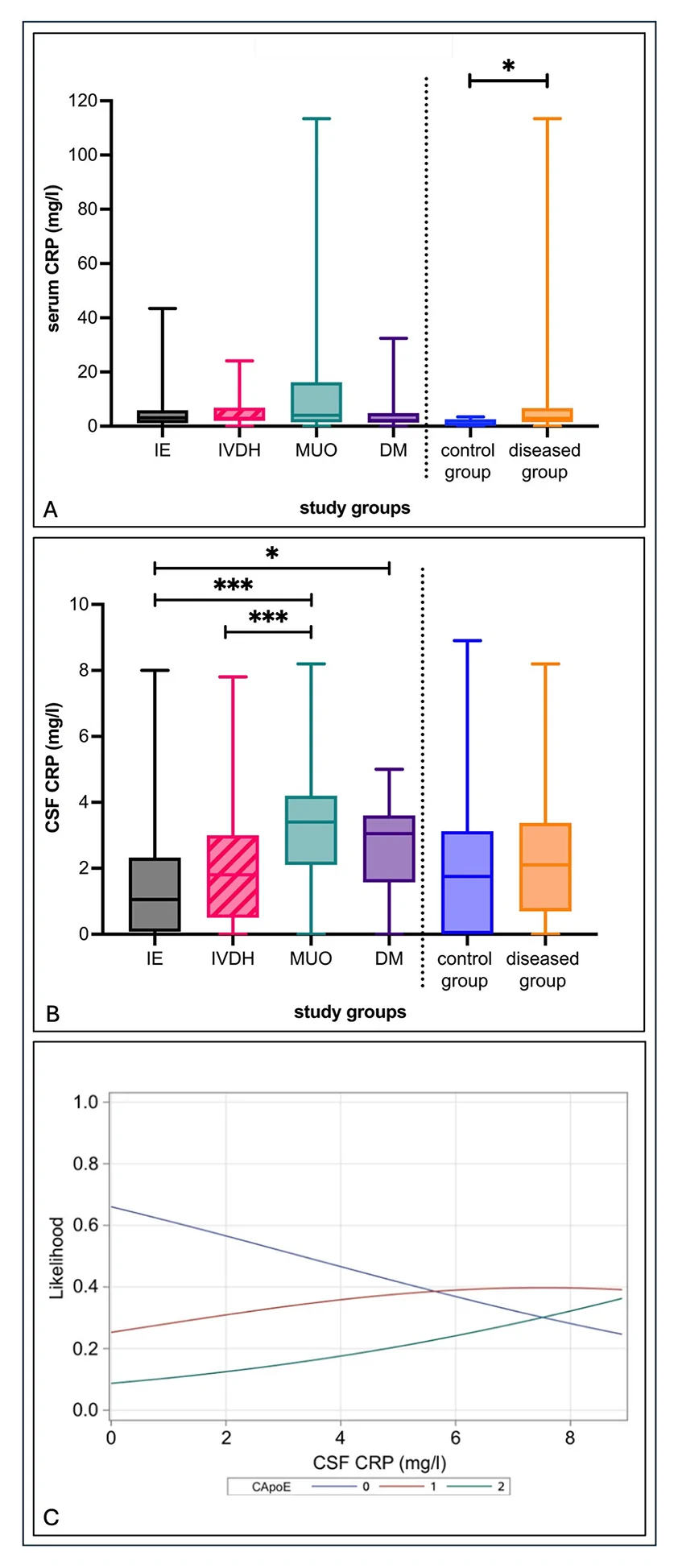
Serum and CSF levels of C-reactive protein (CRP) of healthy control dogs and dogs diagnosed with idiopathic epilepsy (IE), intervertebral disc herniation (IVDH), meningoencephalitis of unknown origin (MUO), or degenerative myelopathy (DM) and their association with CApoE. **(A, B)** Box-and-whisker plots show CRP concentrations in serum **(A)** and CSF **(B)** of the study cohort. The dotted line separates individual disease groups from the healthy controls and the aggregated diseased group. Serum CRP differed significantly between controls and diseased group (*p* = 0.02; Mann-Whitney U test). In CSF, CRP concentrations differed significantly between IE and DM (*p* = 0.03; Kruskal-Wallis test), IE and MUO (*p* = 0.0006) and IVDH and MUO (*p* = 0.008). Panel **(C)** illustrates the estimated probabilities of CApoE (y-axis) in relation to CSF CRP concentrations (x-axis) in all dogs. Lines represent predicted probabilities across the range of CSF CRP values, with color coding corresponding to ApoE concentrations **(C)**: not detectable (0, blue line), C = 0 ng/ml; detectable but not quantifiable (1, red line), C > 0 to < 3.12 ng/ml; and quantifiable (2, green line), C ≥ 3.12 ng/ml. CRP = C-reactive protein. A significant association between CSF CRP and CApoE was observed (*p* = 0.02; OR = 0.81; ordinal logistic regression, Wald test). *p < 0.05; ***p < 0.001.

CRP could be determined in 142 out of 151 serum and CSF samples, excluding the follow up samples of dogs with MUO. The remaining nine samples were excluded due to insufficient volume. Serum CRP levels were higher than those in the CSF in the whole study cohort; however, no significant correlation was observed between serum and CSF CRP concentrations (*n* = 133; r = 0.1648; *p* = 0.06; Spearman correlation). Serum CRP levels differed significantly between diseased dogs and healthy controls (*p* = 0.02, Mann-Whitney U test). After correction for multiple testing, pairwise comparisons showed no statistically significant differences between any individual disease group and healthy controls or among the individual disease subgroups (Figure 3A).

No significant difference in CSF CRP concentrations was observed between diseased dogs and healthy controls (*p* = 0.45; Mann-Whitney U test). In contrast, analysis across all groups, including controls, revealed a significant difference (*p* = 0.0002; Kruskal-Wallis test). *Post hoc* pairwise comparisons using Dunn's test showed that dogs with MUO had significantly higher CSF CRP concentrations than those with IVDH (*p* = 0.008) and IE (*p* = 0.0006). Furthermore, dogs with DM showed higher CSF CRP levels than those with IE and IVDH, with the difference between DM and IE reaching statistical significance (*p* = 0.03) (Figure 3B).

No significant association was found between serum CRP and CApoE (*p* = 0.15; ordinal logistic regression [Wald test]). In contrast, a significant association was identified between CSF CRP and CApoE (*p* = 0.02; OR = 0.81) for the whole study cohort, indicating that higher CSF CRP concentrations were associated with an increased likelihood of classification into a higher CApoE (Figure 3C). This association was particularly evident in dogs with IVDH (*p* = 0.01; OR = 0.579).

### 3.5 Laboratory findings and their association with ApoE

**Figure 4:**
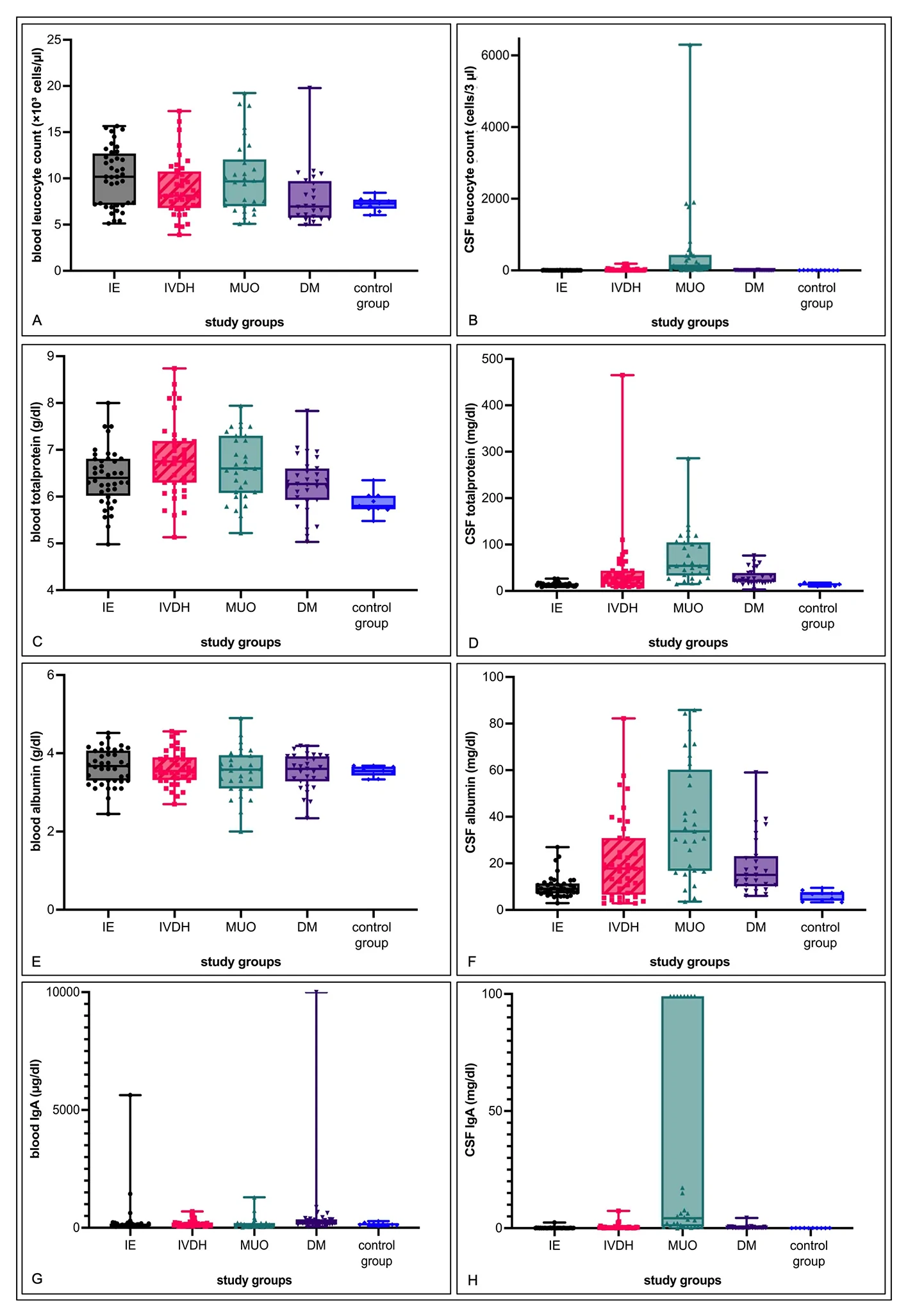
Blood and cerebrospinal fluid (CSF) parameters across study groups. **(A, C, E, G)** show leukocyte count, total protein, albumin, and IgA levels in blood. **(B, D, F, H)** show the corresponding parameters in CSF. Data are presented as box-and-whisker plots indicating the minimum (lower whisker), maximum (upper whisker), median (line within the box), and interquartile range (25th to 75th percentile). IE, idiopathic epilepsy; IVDH, intervertebral disc herniation; MUO, meningoencephalomyelitis of unknown origin; DM, degenerative myelopathy.

To assess whether potential systemic inflammatory changes or specific diseases influenced ApoE levels, laboratory parameters were evaluated and associations calculated. Blood and CSF laboratory parameters across the study groups are presented in Figures 4A–H. Diseased dogs exhibited higher blood leucocyte counts and total protein concentrations than controls (Figures 4A, C). Leukocytosis (>12 × 10^3^/μl) was present in 18.4% of dogs, distributed across all diseased subgroups; hypoproteinemia (< 5.4 g/dl) and hyperproteinemia (>7.5 g/dl) were observed in 5.3% and 6.6%, respectively. Mild and marked serum IgA elevations were detected in 37.7% and 31.9% of dogs. None of the blood parameters, including leucocyte count, total protein, albumin, and IgA, showed a significant association with CApoE for the whole study cohort. In dogs with IE, CApoE was significantly associated with leucocyte count (*p* = 0.02; OR = 0.747; ordinal logistic regression [Wald test]), indicating that higher leucocyte counts in blood were associated with an increased likelihood of classification into a higher CApoE.

In CSF of diseased dogs, all values had the highest mean value in dogs with MUO. Pleocytosis (>15 leucocytes/3 μl) was detected in 30.4%, exclusively in dogs with MUO (*n* = 29) or IVDH (*n* = 14) (Figure 4B). Elevated CSF protein was found in 30.5%, based on reference intervals differing by puncture site. The sample with the highest CSF total protein concentration in the IVDH group was reviewed individually. As no evidence of blood contamination was identified, the value was retained in the analysis. CSF IgA was mildly and markedly elevated in 20.6% and 47.5% of dogs, respectively.

Among healthy controls, serum IgA was elevated in a subset of animals (70% mild, 20% marked). Mild CSF IgA elevation was observed in one dog. All other blood and CSF parameters were within reference ranges.

CApoE was significantly associated with leucocyte count and albumin concentration in CSF (*p* = 0.04; OR = 0.999 and *p* = 0.02; OR = 0.990; ordinal logistic regression [Wald test]) for the whole study cohort. No significant associations were found for the remaining CSF parameters, within the overall study population or across the study groups.

## 4 Discussion

ApoE has been studied intensively for many years in human neurodegenerative diseases, as the ApoE4 isoform is a risk factor for Alzheimer's disease (16, 37, 38). However, information on CSF ApoE concentrations in dogs is still very limited. To the best of authors' knowledge, the present study is the first to investigate CSF ApoE concentrations across various canine CNS diseases. Only one previous study has investigated CSF ApoE concentrations in dogs using methods other than ELISA, reporting concentrations ranging from 6.0 to 11.9 mg/l in healthy mongrel dogs and purebred foxhounds (39).

In the present study, ApoE was detectable in the CSF of approximately 44% of the study population, with concentrations ranging from 3.53 to 99.42 ng/ml. Notably, only one healthy control showed measurable ApoE levels, suggesting that CSF ApoE may be increased in dogs with neurological disease. In contrast, previously reported CSF ApoE concentrations in humans, as determined by ELISA, ranged widely from approximately 1.2 ± 0.7 mg/l in patients with Alzheimer's disease to 13.6 ± 12.1 mg/l in healthy individuals, with epilepsy patients exhibiting approximately half the concentrations observed in healthy controls (40, 41). These differences may reflect species-specific variation in ApoE biology between humans and dogs. Although these studies provide a relevant reference point, direct comparison with our findings is limited by differences in measurement ranges and units, as well as variability in ELISA detection depending on the antibody pairs used (42).

As neuronal injury induces astrocyte activation and neuronal loss, ApoE is secreted primarily by astrocytes and microglia to support synaptogenesis and regeneration (10, 11, 43). Previous studies have demonstrated the presence of perilesional activated astrocytes in MUO (44), which may explain why dogs with MUO exhibited the highest proportion of quantifiable CSF ApoE concentrations in the present study. Dogs with MUO in this study more frequently presented with multifocal lesions, and the presence of lesions at multiple sites may be associated with enhanced astrocyte activation. Based on previous findings increased astrocyte activation could contribute to elevated ApoE secretion into the CSF.

Dogs with IE were more likely to be classified into a higher CApoE level with increasing seizure frequency. Each epileptic seizure induces a localized inflammatory response in the brain, characterized by blood-brain barrier disruption, neuronal cell death, and activation of astrocytes and microglia (30, 45). A high seizure burden may exacerbate these processes, which may cause increased ApoE expression and subsequently elevated CSF ApoE concentrations. We additionally observed an association between peripheral blood leucocyte counts and increased CSF ApoE concentrations in dogs with IE, which may be explained by seizure-induced systemic alterations, including transient postictal leukocytosis (46), and further supports the link between seizure burden and ApoE upregulation.

In dogs with IVDH, only those with ANNPE and HNPE showed quantifiable ApoE concentrations in the CSF. Notably, dogs with ANNPE or HNPE in the present study were presented earlier after onset of clinical signs than dogs with Hansen type I extrusion or Hansen type II protrusion, likely due to the acute and often severe neurological deficits associated with these conditions. Consequently, CSF sampling may have occurred closer to the time of neuronal injury, when ApoE was still detectable within the CSF. In contrast, undetectable or markedly reduced CSF ApoE concentrations in dogs with Hansen type I extrusion or Hansen type II protrusion may reflect redistribution of ApoE from the CSF into interstitial and intracellular CNS compartments over time (10). Within these compartments, ApoE-containing lipoproteins support lipid transport, synaptogenesis, and neuronal regeneration (15, 16, 43). Supporting this hypothesis, studies in murine models and humans demonstrated increased ApoE accumulation in astrocytes and neuropil shortly after ischemic CNS injury, whereas later stages were characterized predominantly by ApoE localization within degenerating neurons and neuronal processes (10). Future studies should investigate whether dogs with Hansen type I extrusion likewise show elevated CSF ApoE concentrations when sampled immediately after onset of clinical signs.

Human studies have demonstrated a role for ApoE and its different genotypes in degenerative neurological disorders such as Alzheimer's disease, whereas its involvement in amyotrophic lateral sclerosis (ALS) remains controversial (14, 47–49). Due to similarities in genetic background, lesion distribution, and clinical presentation, DM and ALS share several pathological features including inflammatory processes, reactive microgliosis, and astrocytic activation although DM is characterized by a more pronounced involvement of the white matter (35, 50, 51). While Lacomblez et al. (52) reported an association between elevated plasma ApoE levels and accelerated disease progression, and reduced survival time in ALS patients, no significant association was identified between disease duration and CSF ApoE concentrations in dogs with DM in the present study. A possible explanation is that, due to the elevated release of proinflammatory cytokines by chronically activated astrocytes and microglia, prolonged exposure to these cytokines induces a potentially neurotoxic activation state in astrocytes, impairing their capacity to secrete ApoE (37, 45, 53). Based on the findings of this study, the precise role of ApoE in DM pathogenesis remains to be elucidated.

Previous studies have demonstrated elevated CSF CRP concentrations in dogs with inflammatory, non-infectious diseases compared with dogs with IE or spinal cord compression (54, 55) which is consistent with the findings of the present study. Important aspects of ApoE are its immunomodulatory properties (13, 23). Contrary to our hypothesis this study demonstrated a significant association between increasing CSF CRP levels and increased detectability of CSF ApoE (*p* = 0.02, Figure 3C), particularly in dogs with IVDH (*p* = 0.01). IVDH induces an inflammatory response in the epidural space at the site of disc extrusion due to nucleus pulposus material (29). Since CRP is an inflammatory mediator, it could serve as a marker for local inflammatory reaction. Following spinal cord injury, activated immune cells such as microglia secrete proinflammatory cytokines, which induce CRP synthesis within the CNS by neurons, microglia, and astrocytes (25–27, 56). Disruption of the blood-spinal cord barrier allows these cytokines to enter the systemic circulation, where they stimulate hepatic CRP production (56, 57). Anderson et al. (58) demonstrated that dogs with severe spinal cord injury following disc herniation had higher CRP levels in the CSF, since peripheral CRP could spill over into the CSF. Furthermore, previous experimental studies have shown that ApoE-deficiency is associated with enhanced inflammatory responses, including increased hepatic CRP concentrations in ApoE-knockout dogs, and increased proinflammatory cytokines expression in ApoE-deficient mice (19, 21, 23). Together, these previous findings suggest that ApoE may play an important role in regulating neuroinflammatory processes. Accordingly, increased ApoE release from astrocytes has been proposed as a potential counter-regulatory response that may attenuate proinflammatory microglial signaling and contribute to neuroprotection (13, 23, 24, 59, 60).

There are some limitations to our study. The healthy control dogs consisted exclusively of Beagles with an age range of 27–36 months, whereas the diseased cohort showed a broader age distribution (7–166 months). This resulted in a homogenous control group and an age discrepancy that may limit the generalizability. However, no significant association between age and CApoE was detected in the present study. Although a statistical power of 81.5% was calculated for this study with 10 healthy control dogs compared to 141 diseased dogs, a larger number of quantifiable ApoE measurements would have further increased the statistical power.

A major analytical limitation of the present study was that only a small proportion of CSF samples (20/151) contained quantifiable ApoE concentrations, whereas most samples were either below the LLOQ or undetectable. In addition, the inter-assay CV exceeded 30%, indicating substantial variability between assay runs and exceeding the generally accepted limits for quantitative bioanalytical assays. In contrast, intra-assay precision was below 10% for both control materials, suggesting good repeatability within individual assay runs. The discrepancy between intra- and inter-assay precision indicates that variability mainly arose from factors influencing different assay runs, such as environmental conditions, operator-dependent handling and reagent stability. Furthermore, a matrix effect cannot be excluded, as preliminary optimization experiments showed increased ApoE concentrations after two-fold dilution of CSF samples, although dilution linearity, recovery, and matrix effects were not formally evaluated.

Taken together, these analytical limitations primarily affect the interpretation of absolute quantitative MApoE concentrations. Therefore, the primary analyses and conclusions of this study were based on the categorical classification of ApoE (CApoE), whereas quantitative MApoE analyses were considered complementary exploratory findings.

The highest MApoE was observed in a dog with DM. As erythrocyte counts were not consistently available, a contribution of blood contamination to this individual measurement cannot be excluded. Exclusion of this single observation did not alter the statistical results, indicating that it did not influence the overall interpretation of the quantitative analyses.

Dogs with MUO, in which lesions are located within the brain parenchyma, exhibited the highest proportion of samples with measurable ApoE levels, whereas dogs with DM—primarily affecting the thoracolumbar spinal cord—had the fewest quantifiable samples. As most CSF samples were collected suboccipitally and the exact sampling site was not consistently documented, differences between lesion location and sampling site may have influenced ApoE concentrations across disease groups, contributing to variability in the data. This effect is likely explained by the fact that CSF collected caudal to a lesion is more markedly altered than CSF obtained cranially, due to its predominant caudal flow (61, 62).

The heterogeneous study population in a clinical setting, characterized by varying types of IVDH, differing intervals between onset of clinical signs and sampling, and substantial variability of clinical manifestations among MUO cases, complicates the evaluation of the diagnostic and clinical utility of ApoE as a potential biomarker. Nevertheless, this investigation should be regarded as an initial exploratory study providing an overview of ApoE across diverse CNS lesion types in dogs.

Further studies are required to determine whether ApoE may represent a potential biomarker for canine CNS disorders, as its diagnostic and prognostic value has not yet been established. The evaluation of ApoE as a potential biomarker should be continued in a follow-up study focusing exclusively on dogs with ANNPE and HNPE and with cluster seizures and/or status epilepticus. In translational research, domesticated dogs have proven to be an ideal model for the study of human diseases, not only due to their pronounced anatomical and physiological similarities to humans, but also because they share the same living environment as their owners, thereby being exposed to comparable environmental conditions (63, 64). Analogies between human and canine epilepsy, as well as between naturally occurring canine IVDH and acute spinal cord injuries in humans, underscore the relevance of the dog as a translational model in biomedical research (65, 66). The more detailed characterization of ApoE and neurological disorders in dogs may therefore not only advance veterinary medicine but also yield valuable insights for human healthcare.

## 5 Conclusion

To the best of the authors' knowledge this study presents the first investigation of ApoE in CNS disorders in dogs and includes an analytical evaluation of an ELISA for ApoE measurement in canine CSF. Dogs with CNS disease were more likely to have detectable ApoE in the CSF than healthy controls. In contrast to findings reported in previous human studies, but consistent with our hypothesis, detectable ApoE was observed predominantly in dogs with severe peracute and acute CNS diseases, potentially reflecting tissue destruction and ApoE release in the CSF. Conversely, ApoE was detected less frequently in chronic degenerative conditions, suggesting its presence in tissues may be associated with ongoing regenerative processes. Contrary to our initial hypothesis, higher ApoE categories were positively associated with CSF CRP concentrations in dogs with IVDH, suggesting a potential role of ApoE in modulating inflammatory processes. Overall, these findings suggest that ApoE may play a role in regeneration and modulation of inflammation in dogs with CNS diseases. However, given the limited number of quantifiable samples and the analytical limitations of the assay, further studies are required to clarify the biological role of ApoE and to evaluate its potential clinical utility.

## Data Availability

The raw data supporting the conclusions of this article will be made available by the authors, without undue reservation.
